# Novel Lycorine Derivatives as Anticancer Agents: Synthesis and *In Vitro* Biological Evaluation

**DOI:** 10.3390/molecules19022469

**Published:** 2014-02-21

**Authors:** Peng Wang, Hui-Hui Yuan, Xue Zhang, Yun-Ping Li, Lu-Qing Shang, Zheng Yin

**Affiliations:** 1 College of Pharmacy & State Key Laboratory of Elemento-Organic Chemistry, Nankai University, Tianjin 300071, China; 2 Department of Chemistry & State Key Laboratory of Elemento-Organic Chemistry, Nankai University, Tianjin 300071, China; 3 College of Life Sciences, Nankai University, Tianjin 300071, China

**Keywords:** lycorine derivatives, anticancer, synthesis

## Abstract

Lycorine, which is the most abundant alkaloid isolated from the *Amaryllidaceae* family of plants, reportedly exhibits promising anticancer activities. Herein, a series of novel lycorine derivatives were synthesized and evaluated for their *in vitro* inhibitory activities against seven different cancer cell lines, including A549, HCT116, SK-OV-3, NCI-H460, K562, MCF-7 and HL-60. The results indicated that compounds bearing diverse amine substituents at the C-2 position demonstrated good anticancer activities. The selectivity towards different cancer cell lines of the synthesized derivatives is discussed.

## 1. Introduction

Cancer is a proliferation disorder disease with apoptosis obstacles. It strikes more than one-third of the World’s population and causes over 20% of all deaths [1]. The standard cancer treatment protocols include surgery, radiotherapy and chemotherapy. Unfortunately, chemotherapy is not effective in treating cancers associated with innate resistance to apoptosis and/or acquired resistance to drugs during treatment [2]. Discovery of novel effective anticancer medicines is therefore of great importance. As a source of drugs, natural products play an important role in drug discovery. Up to 80% of approved anticancer drugs over the time frame from 1981 to 2010 derived from natural products [3,4]. Plant species belonging to the *Amaryllidaceae* family have been well known for centuries to exhibit diverse medicinal properties. Many of these plants have been used historically for the treatment of cancer, since the oil of the daffodil *Narcissus poeticus L.* was used by Hippocrates of Kos [5]. The application of narcissus oil in cancer management continued during the middle ages in China, North Africa and Central America [6]. The major components isolated from the *Amaryllidaceae* plant family are alkaloids. Hundreds of alkaloids have been isolated and extensively studied for their pharmacological properties since 1877 [7]. Lycorine is one of the most common alkaloids within *Amaryllidaceae* family of plants and reportedly exhibits promising anticancer properties [7,8,9,10,11,12].

Lycorine has displayed multiple inhibitory properties towards various cancer cell lines, including lymphoma [7], carcinoma [8], multiple myeloma [9], melanoma [10], leukemia [7,11], human U373 glioblastoma, human A549 non-small-cell-lung cancer, human OE21 esophageal cancer and human Hs683 anaplastic oligodendroglioma cell lines [12]. Previous studies revealed that lycorine exhibited significantly higher antiproliferative activities in cancer cells than in normal cells, and it could be used to combat cancer cells which may or may not be sensitive to proapoptotic stimuli [12]. Further studies provided a mechanistic insight into its anticancer properties. Evdokimov *et al.* reported that lycorine exhibited cytostatic effects by targeting the actin cytoskeleton rather than by inducing apoptosis in cancer cells [13]. However, lycorine was found to induce apoptosis as well as arrest cell cycle of HL-60, KM3, K562, Hey1B cells in different phases [7,14,15,16]. Recently, the inhibitory effect on vasculogenic mimicry of melanoma cells [17] and suppression of neovascularization of ovarian cancer Hey1B cells by lycorine were reported [16]. Many factors thus seem involved in the anticancer properties of lycorine and a clear mechanism remains to be determined. Nevertheless, the anticancer properties of lycorine have drawn significant attention from chemists [5,6,11,12,13]. In the present study we report the synthesis of some novel lycorine derivatives and the evaluation of their *in vitro* anticancer activities.

## 2. Results and Discussion

### 2.1. Chemistry

The hydrochloride salt of lycorine (**1**) is commercially available, but its solubility is poor in most organic solvents. Therefore, 1, 2-diacetyllycorine (**2**) obtained from the acetylation of **1** with Ac_2_O/Py was used as the key intermediate for further modifications.

**Scheme 1:**
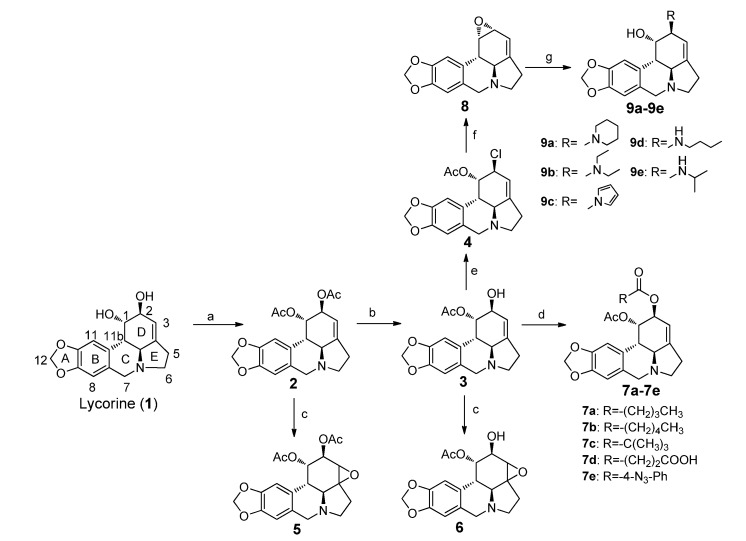
Synthesis of lycorine derivatives.

**Figure 1:**
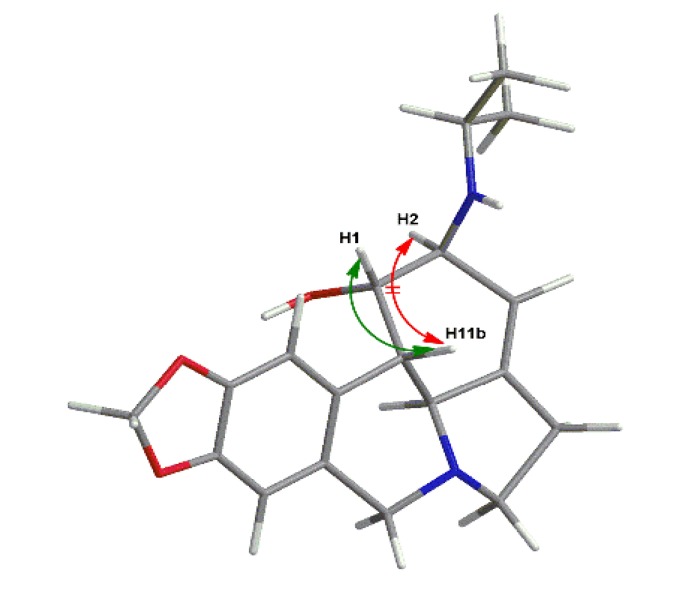
NOESY analysis of **9e**.

Several derivatives were synthesized to investigate the influences of different substituents at the C-2 position. As outlined in the Scheme 1, the intermediate **3** was prepared via the selective deacetylation of the C-2 hydroxyl group using conc. HCl in methanol since the C-2 hydroxyl group is more reactive than the C-1 hydroxyl group [13]. The C-2 hydroxyl of **3** was then coupled with diverse acyl chlorides to afford derivatives **7a**–**e**. Alternatively, the replacement of the C-2 hydroxyl group by chloride using phosphorus oxychloride as halogenating agent provided 1-acetyl-2-chlorolycorine (**4**), which was further treated with sodium methoxide to produce 1,2-α-epoxyllycorine (**8**). Compounds **9a**–**e** were obtained via the opening of the epoxide using the corresponding amines. The nucleophilic attack of the epoxide should occur from the least hindered side with high regioselectivity to afford **9a**–**e**, according to a previous report [18]. This conclusion was confirmed using NOESY analysis. Taking **9b** as an example, a correlation peak was found for H11b/H1, but no correlation between H11b/H2 was observed (Figure 1). Oxidation of the double bond in D-ring with *m*-CPBA led to derivatives **5** and **6**.

*Reagents and conditions*: (a) Ac_2_O, Py; (b) HCl, MeOH; (c) *m*-CPBA, DCM; (d) RCOCl, Py, DCM; (e) POCl_3_, NaCl, HCl; (f) CH_3_ONa, MeOH; (g) RNH_2_ or R_2_NH.

### 2.2. Biological Activity Results

*In vitro* anticancer activities of the synthesized lycorine derivatives were evaluated using the MTT colorimetric assay against a panel of seven human cancer cell lines [19], including non-small-cell-lung cancer (A549), colon carcinoma (HCT116), ovarian carcinoma (SK-OV-3), large-cell-lung cancer (NCI-H460), human myelogenous leukemia (K562), promyelocytic leukemia (HL-60) and human breast adenocarcinoma (MCF-7) cell lines. The results were expressed as IC_50 _for the inhibitory activities and are summarized in Table 1.

**Table 1:** *In vitro* anticancer activities of lycorine derivatives.

No.	cLog P *^a^*	IC_50_/μM *^b^*						
		A549	HCT116	SK-OV-3	NCI-H460	K562	MCF-7	HL-60
**1**	0.39	6.5 ± 0.3	3.0 ± 0.2	3.0 ± 0.3	3.3 ± 0.3	7.5 ± 0.5	3.9 ± 0.2	4.1 ± 0.1
**2**	2.16	20.6 ± 0.8	>20	16.4 ± 0.5	>20	>20	>20	>20
**3**	1.26	>20	9.4 ± 0.5	18.2 ± 0.2	10.6 ± 0.4	18.6 ± 0.3	19.5 ± 0.2	>20
**4**	2.73	>20	>20	>20	>20	>20	>20	>20
**5**	1.54	>20	9.8 ± 1.0	>20	>20	>20	>20	>20
**6**	0.69	>20	>20	>20	>20	>20	>20	>20
**7a**	3.74	>20	10.2 ± 0.6	>20	17.8 ± 0.3	>20	18.1 ± 0.3	>20
**7b**	4.27	19.2 ± 0.5	13.6 ± 1.3	10.1 ± 0.3	13.1 ± 0.4	>20	17.8 ± 0.5	>20
**7c**	3.39	>20	13.5 ± 0.6	16.7 ± 0.5	14.3 ± 0.4	20.9 ± 0.5	19.1 ± 0.3	>20
**7d**	−0.35	>20	>20	>20	>20	>20	>20	>20
**7e**	4.70	18.7 ± 1.1	13.8 ± 0.2	17.2 ± 0.6	19.7 ± 0.6	18.5 ± 0.3	>20	>20
**8**	1.35	6.2 ± 0.4	3.9 ± 0.3	3.3 ± 0.3	2.7 ± 0.1	3.4 ± 0.2	4.5 ± 0.2	2.7 ± 0.3
**9a**	2.48	14.2 ± 0.5	7.9 ± 0.3	4.5 ± 0.3	9.9 ± 0.4	>20	>20	8.3 ± 0.2
**9b**	2.42	15.2 ± 0.6	6.4 ± 0.2	16.8 ± 0.4	8.0 ± 0.4	12.8 ± 0.5	5.2 ± 0.3	>20
**9c**	2.14	>20	>20	15.5 ± 0.3	8.1 ± 0.7	12.0 ± 0.8	20.9 ± 0.6	9.1 ± 0.5
**9d**	2.36	16.3 ± 1.3	10.1 ± 0.1	12.6 ± 0.7	6.5 ± 0.2	6.1 ± 0.3	>20	9.7 ± 0.2
**9e**	1.61	>20	20.5 ± 0.7	13.4 ± 0.2	19.8 ± 0.3	>20	19.7 ± 0.5	>20

*^a^*: cLog P was calculated using ChemBioDraw; *^b^*: Values are the mean of at least three independent experiments.

Evidente *et al*. reported that epoxide **8** demonstrated anticancer activities comparable with lycorine against the A549, OE21, Hs683, U373, SKMEL and B16F10 cell lines [12]. In our study, **8** also presented excellent potency against all tested cell lines. However, compared with **2** and **3**, epoxides **5** and **6** derived from **2** and **3**, respectively, showed much reduced activity (>20 μM) against almost all seven cell lines. The results indicated that an epoxide ring at C3-C4 didn’t benefit the anticancer activities and the presence of the double bond of A-ring appeared to be essential for the potency.

Esters **7a**–**e** were synthesized to investigate the influence of modifications of the C-2 hydroxyl group. In general, **7a**, **7b**, and **7c** exhibited moderate potency against all of tested cell lines, except K562 and HL-60, while **7e** demonstrated moderate potency against all of the tested cell lines except MCF-7 and HL-60. Interesting selectivity was observed with ester substituents as well. Generally, esterification of the C-2 hydroxyl group with aliphatic or aromatic acids slightly increased the activities for the A549 and SK-OV-3 cell lines, but decreased the activities for HCT116, NCI-H460, K562 and MCF-7 at different levels (**2**, **7a**, **7b**, **7c**, **7e** *vs*. **3**). For the HCT116 and NCI-H460 cell lines, both the aliphatic (**2**, **7a**, **7b**, **7c**) and the aromatic substituent (**7e**) at the C-2 position appeared to be tolerated well, but for the K562 cell line, an aromatic substituent (compound **7e**) exhibited better activities than linear aliphatic substituents (**2**, **7a**, **7b**). However, the results were the opposite for the MCF-7 cell line. It is noteworthy that **7d** with a carboxylic acid terminus lost its activity against all of the tested cell lines. The decrease in activities for **7d** might result from the ionic nature of the carboxylate group and the significant change of cLog P. In a previous report [20], cell permeability was an important determinant for anticancer activities of lycorine derivatives. It is likely that this observation reflects the difficulty of cell penetration for highly hydrophilic lycorine derivatives like **7d**. In another variation, the hydroxyl group at the C-2 position was replaced by a chlorine to afford **4**. This replacement led to the dramatic decrease in potency against all of the tested cell lines (>20 μM).

To further examine the influence of substituents at the C-2 position, a variety of amines (alkylamine, piperidine, pyrrole) were introduced to yield **9a**–**e** via the opening of the epoxide of **8**. Overall, these derivatives exhibited higher potency than esters **7a**–**e**. From the perspective of hydrogen-bond acceptor/donor, tertiary amino substituents at the C-2 position of compounds **9a**–**c** could be an acceptor, while the secondary amino substituents of compounds **9d**–**e** could be both an acceptor and a donor. No clearly difference was observed between the activities of **9a**–**c** and **9d**–**e** towards all of tested cell lines. Thus, a hydrogen donor at this position might be less important for the anticancer activities than a hydrogen acceptor. Lycorine exhibited similar good activities towards all of tested cell lines, while amine substituents at the C-2 position improved the selectivity to different cancer cell lines. Aliphatic substituents (compounds **9a**, **9b**, **9d**) exhibited better activities than aromatic substituent (**9c**) for A549 and HCT116 cell lines. For SK-OV-3, **9a** (IC_50_ = 4.5 μM) represented comparable activity with lycorine (IC_50_ = 3.0 μM). The compound with a linear aliphatic substituent **9d** provided the best activities for NCI-H460 (IC_50 _= 6.5 μM) and K562 (IC_50_ = 6.1 μM, which is better than that of lycorine, IC_50_ = 7.5 μM) while the bulky aliphatic substituent derivative 9b demonstrated the best potency against HCT116 (IC_50 _= 6.4 μM) and MCF-7 (IC_50 _= 5.2 μM).

## 3. Experimental

### 3.1. General

All solvents were treated by standard techniques before use. Lycorine hydrochloride (purity > 98%) was purchased from Baoji Guokang Biotechnology Co. Ltd (Baoji, China). Cell culture medium, fetal bovine serum, and trypsin were purchased from Thermo Scientific (Waltham, MA, USA). All reagents were purchased from the commercial suppliers and were used without further purification. ^1^H- and ^13^C-NMR spectra were recorded on a Bruker AVANCE-400 (400 MHz) NMR spectrometer (Billerica, MA, USA) (^1^H, 400 MHz; ^13^C, 100 MHz) in CDCl_3_, CD_3_OD using TMS as an internal standard. Chemical shifts are reported in parts per million δ in ppm and coupling constants are provided in hertz (Hz). ESI-MS spectra were recorded on a Shimadzu LCMS-2020 (Shimadzu, Kyoto, Japan). HRMS were recorded on a Varian 7.0 T high resolution ESI-FTICR mass spectrometer (Palo Alto, CA, USA). Silica gel TLC (GF254) was used to monitor the reactions, which were visualized under UV light at 254 nm and 365 nm. Flash chromatography was performed using silica gel (200–300 mesh).

### 3.2. Cell Lines

The cell lines used in this study were purchased from the Shanghai Cell Bank, Chinese Academy of Medical Sciences (Shanghai, China). All cell lines were maintained in a humidified atmosphere containing 5% CO_2_ at 37 °C. All the cancer cells were cultured in RPMI-1640 medium supplemented with 10% FBS, 100 U/mL penicillin, and 100 μg/mL streptomycin. The cells were seeded into 96 well plates in 100 μL at plating density of 2 × 10^3^ cells per well. After incubation for 24 h, the cells were allowed to attach. The cells were treated with different concentrations of compounds and then incubated for 72 h under the same conditions. Compounds were dissolved in DMSO at stock concentrations of 20 mM.

### 3.3. *In Vitro* Growth Inhibition Assay

The concentrations which were used to calculate the IC_50_ values were: 100, 50, 25, 12.5, 6.25, 3.125, 1.56, 0.78 μM. The stock solutions were diluted with complete medium before use. Untreated cells were used as controls. The cells were then treated for 72 h with varying concentrations of compounds. 20 μL of 3-(4,5 dimethylthia-zol-2-yl)-2,5-diphenyl-tetrazoliumbromide (MTT, Sigma, St. Louis, MO, USA) solution (5 mg/mL in PBS) was added to each well and incubated for 4 h at 37 °C. After the removal of the medium, MTT formazan was dissolved in 150 μL DMSO and monitored using a microplate reader at a wavelength of 570 nm. All experiments were performed in triplicate wells and were repeated at least three times.

### 3.4. Chemistry

*(1S,2S,3a^1^S,12bS)-2,3a^1^,4,5,7,12b-Hexahydro-1H-[1,3]dioxolo[4,5-j]pyrrolo[3,2,1-de]phenanthridine-1,2-diyl diacetate* (*1,2-diacetyllycorine*, **2**). To a solution of lycorine (3.17 g, 11 mmol) in pyridine (8.0 mL) was added acetic anhydride (9.5 mL) and the solution was stirred at 50 °C for 12 h. Subsequently methanol (25 mL) was added. The mixture was stirred at r.t. for 3 h. The solvent was removed under reduced pressure followed by the addition of DCM (50 mL) and water (40 mL). The organic layer was washed with aqueous NaHCO_3_ solution and brine, respectively, and dried over anhydrous Na_2_SO_4_. After filtration, the filtrate was concentrated and the crude residue was purified using a silica gel chromatography and was eluted with DCM/EtOAc/CH_3_OH (10:10:1) to give **2** as a white solid (3.78 g, 93.3%). ^1^H-NMR (400 MHz, CDCl_3_) δ: 6.75 (s, 1H), 6.57 (s, 1H), 5.91 (s, 2H), 5.74 (s, 1H), 5.53 (s, 1H), 5.26 (s, 1H), 4.16 (d, *J* = 14.0 Hz, 1H), 3.53 (d, *J* = 14.0 Hz, 1H), 3.37 (m, 1H), 2.86 (d, *J* = 10.0 Hz, 1H), 2.77 (d, *J* = 10.4 Hz, 1H), 2.65 (m, 2H), 2.40 (m, 1H), 2.08 (s, 3H), 1.95 (s, 3H); ^13^C-NMR (100 MHz, CDCl_3_) δ: 170.0, 169.8, 146.4, 146.2, 129.4, 126.5, 113.8, 107.3, 105.0, 101.0, 70.9, 69.2, 61.2, 56.9, 53.6, 40.5, 28.7, 21.2, 21.0; ESI-MS: *m/z* 372 [M + H]^+^; HRMS: *m/z* calcd. for C_20_H_22_NO_6_ 372.1442 [M + H]^+^; found: 372.1445.

*(1S,2S,3a^1^S,12bS)-2-Hydroxy-2,3a^1^,4,5,7,12b-hexahydro-1H-[1,3]dioxolo[4,5-j]pyrrolo[3,2,1-de]-phenanthridin-1-yl acetate* (*1-acetyllycorine*, **3**). To a solution of **2 **(0.96 g, 2.58 mmol) in methanol (100 mL) was added conc. HCl (20 mL), and the solution was stirred at 55 °C for 1 h. The mixture was made alkaline (pH = 8) with aqueous NaHCO_3_ solution, followed by the addition of DCM (25 mL). The organic layer was washed with brine (25 mL), dried over anhydrous Na_2_SO_4 _and filtered. The filtrate was concentrated under reduced pressure and the crude residue was purified using silica gel chromatography and was eluted with EtOAc/CH_3_OH (10:1) to give **3** as a white solid (0.50 g, 58.9%). ^1^H-NMR (400 MHz, CDCl_3_) δ: 6.66 (s, 1H), 6.57 (s, 1H), 5.92 (s, 2H), 5.61 (s, 1H), 5.54 (s, 1H), 4.18 (s, 1H), 4.25 (d, *J* = 14.4 Hz, 1H), 3.50 (d, *J* = 14.0 Hz, 1H), 3.35 (m, 1H), 2.85 (d, *J* = 10.4 Hz, 1H), 2.74 (d, *J* = 10.4 Hz, 1H), 2.63 (m, 2H), 2.37 (m, 1H), 1.94 (s, 3H); ^13^C-NMR (100 MHz, CDCl_3_) δ: 170.8, 146.4, 146.2, 143.3, 129.1, 127.0, 117.6, 107.3, 104.9, 101.0, 72.7, 69.2, 61.6, 56.8, 53.7, 39.0, 28.5, 21.1; ESI-MS: *m/z* 330 [M + H]^+^; HRMS: *m/z* calcd. for C_18_H_20_NO_5_ 330.1341 [M + H]^+^; found: 330.1344.

*(1S,2S,3a^1^S,12bS)-2-Chloro-2,3a^1^,4,5,7,12b-hexahydro-1H-[1,3]dioxolo[4,5-j]pyrrolo[3,2,1-de]-phenan-thridin-1-yl acetate* (*1-acetyl-2-chlorollycorine*, **4**). To a solution of NaCl (5 mg, 0.1 mmol) in phosphorus oxychloride (2 mL) was added **3** (156 mg, 0.47 mmol), and the mixture was stirred at 30 °C for 1 h, followed by the addition of conc. HCl solution (0.1 mL). After stirred at 30 °C for another 2 h, the mixture was poured into ice water, made alkaline (pH = 8) with aqueous ammonia solution and was extracted with DCM (25 mL). The organic layer was dried over anhydrous Na_2_SO_4_ and filtered. The filtrate was concentrated under reduced pressure and the crude residue was purified using silica gel chromatography and eluted with EtOAc/CH_3_OH (10:1) to give **4** as a white solid (132 mg, 80.9%). ^1^H-NMR (400 MHz, CDCl_3_) δ: 6.72 (s, 1H), 6.58 (s, 1H), 5.92 (s, 2H), 5.88 (s, 1H), 5.55 (s, 1H), 4.06 (s, 1H), 4.15 (d, *J* = 14.4 Hz, 1H), 3.55 (d, *J =* 14.0 Hz, 1H), 3.37 (m, 1H), 3.18 (d, *J* = 10.4 Hz, 1H), 2.87 (d, *J* = 10.0 Hz, 1H), 2.66 (m, 2H), 2.42 (m, 1H), 1.96 (s, 3H); ^13^C-NMR (100 MHz, CDCl_3_) δ: 170.2, 146.5, 146.4, 144.0, 129.4, 126.5, 116.2, 107.4, 104.8, 101.0, 71.8, 61.3, 56.9, 56.7, 53.6, 38.4, 28.7, 20.9; ESI-MS: *m/z* 348 [M + H]^+^; HRMS: *m/z* calcd. for C_18_H_19_ClNO_4_ 348.0997 [M + H]^+^; found: 348.0990.

*(1a^1^S,10bS,11S,12R)-1a^1^,2,3,5,10b,11,12,12a-Octahydro-[1,3]dioxolo[4,5-j]oxireno[2,3-c]pyrrolo-[3,2,1-de]phenanthridine-11,12-diyl diacetate* (*1,2-diacetyl-3,4-epoxyllycorine*, **5**). To a solution of **2** (371 mg, 1 mmol) in 5 mL DCM was added *m*-CPBA (260 mg, 1.5 mmol), and the mixture was stirred at r.t. for 1 h. After solvent evaporation, the crude residue was purified using a silica gel chromatography eluted with DCM/CH_3_OH (3:1) to give **5** as a white solid (324 mg, 83.7%). ^1^H-NMR (400 MHz, CD_3_OD) δ: 6.76 (s, 1H), 6.59 (s, 1H), 5.86 (s, 2H), 5.82 (s, 1H), 5.67 (s, 1H), 5.20 (s, 1H), 4.74 (d, *J* = 15.2 Hz, 1H), 4.48 (d, *J* = 14.8 Hz, 1H), 4.13 (d, *J* = 11.6 Hz, 1H), 3.83 (m, 1H), 3.49 (d, *J* = 11.6 Hz, 1H), 2.96 (m, 1H), 2.79 (m, 1H), 1.99 (s, 3H), 1.83 (s, 3H); ^13^C-NMR (100 MHz, CD_3_OD) δ: 171.3, 171.2, 149.0, 148.8, 141.3, 125.9, 125.7, 119.0, 108.6, 106.0, 103.0, 72.5, 70.7, 70.0, 69.0, 68.6, 35.7, 27.6, 20.9, 20.6; ESI-MS: *m/z* 388 [M + H]^+^; HRMS: *m/z* calcd. for C_20_H_22_NO_7_ 388.1391 [M + H]^+^; found: 388.1386.

*(1a^1^S,10bS,11S,12R)-12-Hydroxy-1a^1^,2,3,5,10b,11,12,12a-octahydro-[1,3]dioxolo[4,5-j]oxireno[2,3-c]pyrrolo[3,2,1-de]phenanthridin-11-yl acetate (1-acetyl-3,4-epoxyllycorine,* **6**). To a solution of **3** (200 mg, 0.61 mmol) in DCM (4 mL) was added *m*-CPBA (210 mg, 1.22 mmol), and the mixture was stirred at r.t. for 1 h. After solvent evaporation, the crude residue was purified using silica gel chromatography and eluted with DCM/CH_3_OH (3:1) to give **6** as a white solid (153 mg, 72.7%). ^1^H-NMR (400 MHz, CD_3_OD) δ: 6.78 (s, 1H), 6.63 (s, 1H), 5.90 (s, 2H), 5.78 (s, 1H), 5.73 (s, 1H), 4.77 (d, *J* = 15.2 Hz, 1H), 4.52 (d, *J* = 15.2 Hz, 1H), 4.13 (s, 1H), 4.12 (d, *J* = 10.4 Hz, 1H), 3.84 (m, 2H), 3.52 (d, *J* = 11.2 Hz, 1H), 2.98 (m, 1H), 2.82 (m, 1H), 1.86 (s, 3H); ^13^C-NMR (100 MHz, CD_3_OD) δ: 171.7, 148.9, 148.6, 137.9, 126.7, 125.6, 122.7, 108.5, 105.6, 102.9, 72.8, 72.6, 69.3, 69.0, 68.6, 34.4, 27.37, 20.7; ESI-MS: *m/z* 346 [M + H]^+^; HRMS: *m/z* calcd. for C_18_H_20_NO_6_ 346.1258 [M + H]^+^; found: 346.1257.

#### 3.4.1. General Procedure for **7**

To a solution of **3** (1 equiv.) in anhydrous pyridine (2 mL) was added acyl chloride or anhydride (1.2 equiv.) in anhydrous DCM (5 mL) over 15 min at 0 °C, and the solution was stirred at 0 °C for another 3 h. Subsequently, DCM and water were added, and the organic layer was washed using aqueous NaHCO_3_ solution and brine, dried over anhydrous Na_2_SO_4_ and filtered. The filtrate was concentrated under reduced pressure and the crude residue was purified using a silica gel chromatography to give **7a**–**e**.

*(1S,2S,3a^1^S,12bS)-1-Acetoxy-2,3a^1^,4,5,7,12b-hexahydro-1H-[1,3]dioxolo[4,5-j]pyrrolo[3,2,1-de]-phenanthridin-2-yl pentanoate* (*1-acetyl-2-valeryllycorine*, **7a**). Following the previously described procedure, 95 mg (0.28 mmol) of **3** gave **7a** as a pale solid (163 mg, 64.7%). ^1^H-NMR (400 MHz, CDCl_3_) δ: 6.75 (s, 1H), 6.57 (s, 1H), 5.92 (s, 2H), 5.73 (s, 1H), 5.53 (s, 1H), 5.26 (s, 1H), 4.16 (d, *J* = 14.1 Hz, 1H), 3.55 (d, *J* = 14.0 Hz, 1H), 3.38 (dt, *J* = 9.1, 4.7 Hz, 1H), 2.89 (d, *J* = 10.5 Hz, 1H), 2.81 (d, *J* = 10.5 Hz, 1H), 2.66 (s, 2H), 2.43 (dd, *J* = 17.5, 8.7 Hz, 1H), 2.33 (td, *J* = 7.4, 3.3 Hz, 2H), 1.95 (s, 3H), 1.61 (dd, *J* = 15.2, 7.6 Hz, 2H), 1.36 (dd, *J* = 15.0, 7.4 Hz, 3H), 0.91 (t, *J* = 7.3 Hz, 3H); ^13^C-NMR (100 MHz, CDCl_3_) δ: 172.5, 170.0, 146.5, 146.3, 145.8, 129.3, 126.6, 114.0, 107.3, 105.1, 101.0, 70.6, 69.2, 61.2, 56.7, 53.6, 40.4, 34.1, 28.7, 26.9, 22.2, 20.9, 13.7; ESI-MS: *m/z* 414 [M + H]^+^; HRMS: *m/z* calcd. for C_23_H_28_NO_6_ 414.1917 [M + H]^+^; found: 414.1905.

*(1S,2S,3a^1^S,12bS)-1-Acetoxy-2,3a^1^,4,5,7,12b-hexahydro-1H-[1,3]dioxolo[4,5-j]pyrrolo[3,2,1-de]-phenanthridin-2-yl hexanoate* (*1-acetyl-2-hexanoyllycorine*, **7b**). Following the previously described procedure, 95 mg (0.28 mmol) of **3** gave **7b** as a white solid (99 mg, 75.0%). ^1^H-NMR (400 MHz, CDCl_3_) δ: 6.71 (s, 1H), 6.54 (s, 1H), 5.88 (s, 2H), 5.70 (s, 1H), 5.49 (s, 1H), 5.23 (s, 1H), 4.13 (d, *J* = 14.0 Hz, 1H), 3.50 (d, *J* = 14.0 Hz, 1H), 3.34 (m, 1H), 2.85 (d, *J* = 10.0 Hz, 1H), 2.75 (d, *J* = 10.4 Hz, 1H), 2.62 (m, 2H), 2.37 (m, 1H), 2.29 (m, 2H), 1.92 (s, 3H), 1.61 (m, 2H), 1.28 (m, 4H), 0.86 (m, 3H); ^13^C-NMR (100 MHz, CDCl_3_) δ: 172.5, 170.0, 146.4, 146.3, 145.9, 129.4, 126.5, 114.0, 107.3, 105.0, 101.0, 70.6, 69.2, 61.2, 56.9, 53.6, 40.5, 34.3, 31.2, 28.7, 24.6, 22.3, 21.0, 14.0; ESI-MS: *m/z* 428 [M + H]^+^; HRMS: *m/z* calcd. for C_24_H_30_NO_6_ 428.2073 [M + H]^+^; found: 428.2067.

*(1S,2S,3a^1^S,12bS)-1-Acetoxy-2,3a^1^,4,5,7,12b-hexahydro-1H-[1,3]dioxolo[4,5-j]pyrrolo[3,2,1-de]-phenanthridin-2-yl pivalate* (*1-acetyl-2-pivaloyllycorine*, **7c**). Following the previously described procedure, 95 mg (0.28 mmol) of **3** gave **7c** as a pale solid (64mg, 56%). ^1^H-NMR (400 MHz, CDCl_3_) δ: 6.77 (s, 1H), 6.59 (s, 1H), 5.93 (s, 2H), 5.72 (s, 1H), 5.52 (s, 1H), 5.24 (s, 1H), 4.19 (d, *J* = 14.2 Hz, 1H), 3.56 (d, *J* = 14.1 Hz, 1H), 3.41 (dt, *J* = 9.0, 4.7 Hz, 1H), 2.90 (d, *J* = 10.4 Hz, 1H), 2.81 (d, *J* = 10.6 Hz, 1H), 2.68 (s, 2H), 2.44 (m, 1H), 1.97 (s, 3H), 1.22 (s, 9H); ^13^C-NMR (100 MHz, CDCl_3_) δ: 177.2, 169.9, 146.5, 146.3, 145.8, 129.3, 126.6, 113.9, 107.3, 105.1, 101.0, 70.6, 69.1, 61.3, 56.8, 53.6, 40.6, 38.7, 28.6, 27.1, 20.9; ESI-MS: *m/z* 414 [M + H]^+^; HRMS: *m/z* calcd. for C_23_H_28_NO_6_ 414.1917 [M + H]^+^; found: 414.1912.

*4-(((1S,2S,3a^1^S,12bS)-1-Acetoxy-2,3a^1^,4,5,7,12b-hexahydro-1H-[1,3]dioxolo[4,5-j]pyrrolo[3,2,1-de]-phenanthridin-2-yl)oxy)-4-oxobutanoic acid* (*1-acetyl-2-(4-carboxyl-propionate)lycorine*, **7d**). Following the previously described procedure, 150 mg (0.46 mmol) of **3 **gave **7d** as a white solid (178mg, 90.5%). ^1^H-NMR (400 MHz, CDCl_3_) δ: 6.72 (s, 1H), 6.62 (s, 1H), 5.92 (s, 2H), 5.76 (s, 1H), 5.58 (s, 1H), 5.27 (s, 1H), 4.19 (d, *J* = 14.0 Hz, 1H), 3.81 (d, *J* = 14.0 Hz, 1H), 3.50 (brs, 1H), 3.21 (d, *J* = 10.8 Hz, 1H), 3.02 (d, *J* = 10.4 Hz, 1H), 2.80 (m, 1H), 2.74 (m, 2H), 2.54–2.60 (m, 4H), 1.96 (s, 3H); ^13^C-NMR (100 MHz, CDCl_3_) δ: 175.7, 171.3, 169.8, 147.1, 146.7, 143.7, 127.4, 126.4, 115.2, 107.6, 105.0, 101.2, 70.0, 68.4, 60.7, 55.0, 53.2, 38.5, 29.9, 29.8, 28.7, 20.9; ESI-MS: *m/z* 428 [M − H]^−^; HRMS: *m/z* calcd. for C_22_H_24_NO_8_ 430.1502 [M + H]^+^; found: 430.1489.

*(1S,2S,3a^1^S,12bS)-1-Acetoxy-2,3a^1^,4,5,7,12b-hexahydro-1H-[1,3]dioxolo[4,5-j]pyrrolo[3,2,1-de]phenanthridin-2-yl 4-azidobenzoate* (*1-acetyl-2-(4-azidobenzoyl)lycorine*, **7e**). Following the previously described procedure described above, 115 mg (0.35 mmol) of **3** gave **7e** as a white solid (120 mg, 72.0%). ^1^H-NMR (400 MHz, CDCl_3_) δ: 8.02 (d, *J* = 6.8, 2H), 7.02 (d, *J* = 6.8, 2H), 6.77 (s, 1H), 6.63 (s, 1H), 5.92 (s, 1H), 5.91 (s, 2H), 5.68 (s, 1H), 5.53 (s, 1H), 4.23 (d, *J* = 14.0, 1H), 3.72 (d, *J* = 14.0, 1H), 3.47 (m, 1H), 3.14 (d, *J* = 10.8, 1H), 3.09 (d, *J* = 10.8, 1H), 2.74 (m, 2H), 2.68 (m, 1H), 2.00 (s, 3H); ^13^C-NMR (100 MHz, CDCl_3_) δ: 170.0, 164.4, 146.8, 146.6, 145.4, 145.0, 144.3, 131.7, 128.5, 126.5, 126.3, 118.8, 118.6, 114.5, 107.5, 105.0, 101.1, 70.9, 68.9, 61.0, 56.0, 53.5, 39.7, 28.8, 21.0; ESI-MS: *m/z* 475 [M + H]^+^; HRMS: *m/z* calcd. for C_25_H_23_N_4_O_6_ 475.1612 [M + H]^+^; found: 475.1610.

*(1aR,2a^1^S,11bS,11cS)-2a^1^,3,4,6,11b,11c-Hexahydro-1aH-[1,3]dioxolo[4,5-j]oxireno[2,3-a]pyrrolo-[3,2,1-de]phenanthridine* (*1,2-α-epoxylycorine*, **8**). To a solution of **4** (500 mg, 1.44 mmol) in 100 mL of CH_3_OH was added 1.0 g of CH_3_ONa, and the mixture was stirred in ice bath for 20 min. DCM and water was added, and the organic layer was dried over anhydrous Na_2_SO_4_. After filtration, the filtrate was concentrated under reduced pressure and the crude residue was purified using a silica gel chromatography eluted with EtOAc to give **8** as a white solid (166 mg, 42.9%). ^1^H-NMR (400 MHz, CDCl_3_) δ: 7.04 (s, 1H), 6.60 (s, 1H), 5.94 (d, *J* = 4.4 Hz, 2H), 5.76 (d, *J* = 1.6 Hz, 1H), 4.08 (d, *J* = 14.0 Hz, 1H), 3.96 (d, *J* = 4.0 Hz, 1H), 3.58 (d, *J* = 14.0 Hz, 1H), 3.51 (t, *J* = 4.0 Hz, 1H), 3.21 (m, 1H), 2.80 (t, *J* = 13.6 Hz, 2H), 2.61 (m, 1H), 2.43 (m, 1H); ^13^C-NMR (100 MHz, CDCl_3_) δ: 147.9, 146.4, 146.2, 129.5, 129.2, 112.6, 107.5, 105.4, 101.0, 63.1, 57.0, 54.5, 54.0, 49.5, 40.8, 29.3; ESI-MS: *m/z* 270 [M + H]^+^; HRMS: *m/z* calcd. for C_16_H_16_NO_3_ 270.1125 [M + H]^+^; found: 270.1130.

#### 3.4.2. General Procedure for the Preparation of **9**

Compound **8** was dissolved in amine (5 mL, piperidine, diethylamine, pyrrole, *n*-butylamine, isopropylamine for **9a**–**e**, respectively), and the solution was heated to reflux overnight. The amine was removed under reduced pressure and the crude residue was purified using silica gel chromatography to give the products.

*(1S,2S,3a^1^S,12bS)-2-(Piperidin-1-yl)-2,3a^1^,4,5,7,12b-hexahydro-1H-[1,3]dioxolo[4,5-j]pyrrolo[3,2,1-de]phenanthridin-1-ol (2-piperidinelycorine*, **9a**). Following the previously described procedure, 175 mg (0.65 mmol) of **8** gave **9a** as a white solid (102 mg, 44.0%). ^1^H-NMR (400 MHz, CDCl_ 3_) δ: 6.89 (s, 1H), 6.61 (s, 1H), 5.93 (d, *J* = 4.8 Hz, 2H), 5.47 (m, 1H), 4.73 (d, *J* = 2.0 Hz, 1H), 4.13 (d, *J* = 14.0 Hz, 1H), 3.52 (d, *J* = 14.0 Hz, 1H), 3.32–3.36 (m, 2H), 2.70–2.74 (m, 2H), 2.62–2.65 (m, 3H), 2.47–2.50 (m, 3H), 2.37 (m, 2H), 1.55–1.59 (m,4H), 1.45 (m, 2H); ^13^C-NMR (100 MHz, CDCl_3_) δ: 146.6, 146.3, 142.3, 130.4, 127.5, 117.8, 107.9, 104.8, 101.0, 69.1, 65.2, 60.7, 56.9, 53.7, 50.6, 44.4, 28.7, 26.6, 24.5; ESI-MS: *m/z* 355 [M + H]^+^; HRMS: *m/z* calcd. for C_21_H_27_N_2_O_3 _355.2016 [M + H]^+^; found: 355.2015.

*(1S,2S,3a^1^S,12bS)-2-(Diethylamino)-2,3a^1^,4,5,7,12b-hexahydro-1H-[1,3]dioxolo[4,5-j]pyrrolo[3,2,1-de]phenanthridin-1-ol (2-diethylaminelycorine,* **9b**). Following the previously described procedure, 200 mg (0.74 mmol) of **8** gave **9b** as a gray solid (143 mg, 56.5%). ^1^H-NMR (400 MHz, CDCl_3_) δ: 6.88 (s, 1H), 6.59 (s, 1H), 5.93 (d, *J* = 11.2 Hz, 2H), 5.45 (s, 1H), 4.83 (s, 1H), 4.13 (d, *J* = 14.0 Hz, 1H), 3.91 (s, 1H), 3.58 (d, *J* = 14.0 Hz, 1H), 3.36 (m, 1H), 2.80 (m, 5H), 2.67 (brs, 2H), 2.55–2.38 (m, 2H), 1.25 (t, *J* = 7.1 Hz, 6H); ^13^C-NMR (100 MHz, CDCl_3_) δ: 146.6, 146.3, 145.8, 129.7, 127.1, 113.0, 107.5, 105.5, 101.0, 66.8, 65.1, 60.6, 56.5, 53.4, 45.0, 43.9, 29.0, 12.3; ESI-MS: *m/z* 343 [M + H]^+^; HRMS: *m/z* calcd. for C_20_H_27_N_2_O_3_ 343.2016 [M + H]^+^; found:343.2016.

*(1S,2S,3a^1^S,12bS)-2-(1H-Pyrrol-1-yl)-2,3a^1^,4,5,7,12b-hexahydro-1H-[1,3]dioxolo[4,5-j]pyrrolo[3,2,1-de]phenanthridin-1-ol (2-pyrrolelycorine,* **9c**). Following the previously described procedure, 200 mg (0.74 mmol) of **8** gave **9c** as a gray solid (161 mg, 65.3%). ^1^H-NMR (400 MHz, CDCl_3_) δ: 6.62 (s, 1H), 6.58 (s, 1H), 6.56 (s, 1H), 6.10 (s, 1H), 5.98 (s, 1H), 5.88 (d, *J* = 5.2 Hz, 2H), 5.50 (s, 1H), 4.49 (s, 1H), 4.15 (d, *J* = 14.9 Hz, 1H), 3.77 (s, 1H), 3.51 (d, *J* = 15.2 Hz, 1H), 3.35–3.27 (m, 1H), 2.88 (d, *J* = 10.6 Hz, 1H), 2.77 (d, *J* = 10.6 Hz, 1H), 2.67 (m, 2H), 2.44–2.32 (m, 1H); ^13^C-NMR (100 MHz, CDCl_3_) δ: 146.6, 146.3, 139.8, 132.5, 127.8, 117.2, 116.6, 108.3, 107.6, 106.0, 104.8, 101.0, 71.8, 61.1, 57.0, 53.9, 45.3, 41.0, 28.5; ESI-MS: *m/z* 337 [M + H]^+^; HRMS: *m/z* calcd. for C_20_H_21_N_2_O_3_ 337.1547 [M + H]^+^; found:337.1543.

*(1S,2S,3a^1^S,12bS)-2-(Butylamino)-2,3a^1^,4,5,7,12b-hexahydro-1H-[1,3]dioxolo[4,5-j]pyrrolo[3,2,1-de]phenanthridin-1-ol (2-butylaminelycorine,* **9d**). Following the previously described procedure described above, 143 mg (0.36 mmol) of **8** gave **9d** as a pale yellow solid (132 mg, 72.8%). ^1^H-NMR (400 MHz, CDCl_3_) δ: 6.85 (s, 1H), 6.58 (s, 1H), 5.92 (d, *J* = 5.6 Hz, 2H), 5.48 (s, 1H), 4.50 (s, 1H), 4.13 (d, *J* = 14.4 Hz, 1H), 3.48 (d, *J* = 14.0 Hz, 1H), 3.33 (m, 1H), 3.24 (s, 1H), 2.74 (m, 3H), 2.60 (m, 3H), 2.32 (m, 1H), 1.44 (m, 2H), 1.34 (m, 2H), 0.92 (t, *J* = 7.2 Hz, 3H); ^13^C-NMR (100 MHz, CDCl_3_) δ: 146.5, 146.2, 141.5, 130.4, 128.0, 117.6, 107.7, 104.7, 100.9, 69.8, 61.6, 61.2, 57.2, 53.9, 48.3, 41.7, 32.6, 28.6, 20.4, 14.0; ESI-MS: *m/z* 343 [M + H]^+^; HRMS: *m/z* calcd. for C_20_H_27_N_2_O_3_ 343.2016 [M + H]^+^; found: 343.2022.

*(1S,2S,3a^1^S,12bS)-2-(Isopropylamino)-2,3a^1^,4,5,7,12b-hexahydro-1H-[1,3]dioxolo[4,5-j]pyrrolo-[3,2,1-de]phenanthridin-1-ol (2-isopropylaminelycorine,* **9e**). Following the previously described procedure, 160 mg (0.59 mmol) of **8** gave **9e** as a pale yellow solid (175 mg, 90.4%). ^1^H-NMR (400 MHz, CDCl_3_) δ: 6.85 (s, 1H), 6.57 (s, 1H), 5.91 (d, *J* = 5.2 Hz, 2H), 5.45 (s, 1H), 4.45 (s, 1H), 4.12 (d, *J* = 14.4 Hz, 1H), 3.48 (d, *J* = 14.0 Hz, 1H), 3.31 (m, 1H), 3.29 (s, 1H), 3.01 (m, 1H), 2.72 (d, *J* = 10.8 Hz, 1H), 2.60 (d, *J* = 10.8 Hz, 1H), 2.58 (m, 2H), 2.31 (m, 1H), 1.08 (s, 3H), 1.06 (s, 3H); ^13^C-NMR (100 MHz, CDCl_3_) δ: 145.5, 145.1, 139.9, 129.3, 127.1, 116.8, 106.6, 103.7, 99.9, 69.8, 60.1, 57.7, 56.2, 52.9, 46.4, 40.4, 27.6, 22.5, 22.2; ESI-MS: *m/z* 329 [M + H]^+^; HRMS: *m/z* calcd. for C_19_H_25_N_2_O_3_ 329.1860 [M + H]^+^; found: 329.1855.

## 4. Conclusions

A series of novel lycorine derivatives were synthesized and their *in vitro* anticancer activities were evaluated. Diverse amine substituents were introduced into the C-2 position of lycorine for the first time and this series of derivatives exhibited good anticancer properties towards seven cell lines. This provides new opportunities for the investigation of novel lycorine derivatives as anticancer agents. Interestingly, selectivity towards different cell lines with this series of lycorine derivatives was observed. Compounds with different selectivity profiles could potentially be used as probes to study the differences between cell lines.
